# Major mistakes and errors in the use of Trial Sequential Analysis in systematic reviews or meta-analyses – protocol for a systematic review

**DOI:** 10.1186/s13643-022-01987-4

**Published:** 2022-06-04

**Authors:** Christian Gunge Riberholt, Markus Harboe Olsen, Joachim Birch Milan, Christian Gluud

**Affiliations:** 1 Department of Neurorehabilitation, Traumatic Brain Injury, Copenhagen University Hospital − Rigshospitalet, Kettegård Alle 30, 2650 Hvidovre, Denmark; 2 Department of Neuroanaesthesiology, Neuroscience Centre, Copenhagen University Hospital − Rigshospitalet, Blegdamsvej 9, 2100 Copenhagen, Denmark; 3 Copenhagen Trial Unit, Centre for Clinical Intervention Research, The Capital Region, Copenhagen University Hospital − Rigshospitalet, Blegdamsvej 9, 2100 Copenhagen, Denmark

**Keywords:** Meta analysis, Methodology, Systematic review, Trial Sequential Analysis

## Abstract

**Background:**

Adequately conducted systematic reviews with meta-analyses are considered the highest level of evidence and thus directly defines many clinical guidelines. However, the risks of type I and II errors in meta-analyses are substantial. Trial Sequential Analysis is a method for controlling these risks. Erroneous use of the method might lead to research waste or misleading conclusions.

**Methods:**

The current protocol describes a systematic review aimed to identify common and major mistakes and errors in the use of Trial Sequential Analysis by evaluating published systematic reviews and meta-analyses that include this method. We plan to include all studies using Trial Sequential Analysis published from January 2018 to January 2022, an estimated 400 to 600 publications. We will search Medical Literature Analysis and Retrieval System Online and the Cochrane Database of Systematic Reviews, including studies with all types of participants, interventions, and outcomes. Two independent reviewers will screen titles and abstracts, include relevant full text articles, extract data from the studies into a predefined checklist, and evaluate the methodological quality of the study using the AMSTAR 2, assessing the methodological quality of the systematic reviews.

**Discussion:**

This protocol follows the Preferred Reporting Items for Systematic Reviews and Meta-Analysis Protocols (PRISMA-P). The identified mistakes and errors will be published in peer reviewed articles and form the basis of a reviewed guideline for the use of Trial Sequential Analysis. Appropriately controlling for type I and II errors might reduce research waste and improve quality and precision of the evidence that clinical guidelines are based upon.

**Supplementary Information:**

The online version contains supplementary material available at 10.1186/s13643-022-01987-4.

## Introduction

Adequately conducted systematic reviews with meta-analyses are considered the highest level of evidence within evidence-based clinical practice [1–3]. Despite their place at the top of the hierarchical research pyramid, conventional meta-analyses are still at risks of type I errors (*alpha*) due to results reaching significance by chance and type II errors (*beta*) due to results not reaching significance even when an effect exists [1, 4, 5]. The risk of these errors is generally accepted at consensus-based levels (typically 5% for type I and 10% or 20% for type II errors), but may increase beyond those levels due to publication bias, biased trial designs, data heterogeneity, and poorly conducted or inadequately powered meta-analyses with multiple significance testing [1, 4, 5]. The investigated effect of a meta-analysis can reach significance even though the effect might be so small that it is not clinically relevant [6].

Several tools exist for controlling type I and type II errors as described by the Cochrane Handbook [2]. However, little emphasis has been put on mitigating the purely random causes of type I and II errors [7]. As an example, correction for multiplicity issues due to use of several outcomes has historically been under prioritised and underpowered reviews are very common [5, 8–10]. Moreover, there is an increased risk of an exaggerated intervention benefit in small trials due to reporting bias or methodological flaws [11]. In a meta-analytic setting, heterogeneity needs to be adequately examined and considered when designing the Trial Sequential Analysis [5, 11–13].

Trial Sequential Analysis can be used to estimate the diversity-adjusted required information size (DARIS or the ‘meta-analytic sample size’) in random-effects meta-analysis [14]. Trial Sequential Analysis may establish when firm evidence is reached for an effect of an intervention [12–15]. Furthermore, Trial Sequential Analysis can establish futility boundaries and thus indicate when conclusion of no effect can be drawn well before reaching the DARIS [12–15]. If adequate power is not reached by the meta-analysis, DARIS may guide the scaling of future trials [13].

Trial Sequential Analysis can be used to assess imprecision with Grading of Recommendations Assessment, Development and Evaluation (GRADE) [16]. By calculating the DARIS, and compare that with the accrued information, the reviewers can determine if and how to downgrade GRADE for imprecision (see below) [17, 18]. Also, the analysis can supply the reviewers with a *trial sequential analysis-adjusted confidence interval* to demonstrate a realistic confidence interval [15].

To date, numerous systematic reviews and meta-analyses have used Trial Sequential Analysis since it was first presented at the beginning of this millennium [19]. As with all methods, the Trial Sequential Analysis can be misused and misinterpreted. A rigorous process starting when writing the protocol through to the reporting phase of the results in the review is necessary. Predefined parameters such as alpha level, beta level (and power), relative risk reduction, minimally relevant clinical difference, and heterogeneity can to a large extent affect the results of the analysis and should therefore be enclosed in pre-published or registered protocols prior to searching for literature for the systematic review. Failure to do so might ultimately alter the conclusion of the meta-analysis and thereby directly misguide clinical practice [5, 15, 20].

### Objective

In this review, we aim to systematically evaluate the use of Trial Sequential Analysis in published systematic reviews and meta-analyses. Specifically, we seek to evaluate how the authors prepared and conducted their Trial Sequential Analysis, and interpreted their results in the assessment of imprecision in the obtained meta-analytic results. We want to identify the most common major mistakes and errors in order to publish these in peer reviewed journals and update recommendations for a more proper use of the Trial Sequential Analysis programme in future systematic reviews [21]. The Trial Sequential Analysis programme is freely accessible from www.ctu.dk/tsa in a java-format and compatible with RevMan 5.0 [15, 20, 22].

## Methods

### Criteria for considering studies for this quality assessment study

As this is a methodological review examining the use of Trial Sequential Analysis in systematic reviews or in meta-analyses, there are only a few criteria for considering eligible reviews. This protocol adheres to the reporting guidelines PRISMA-P (Supplemental material) [23].

#### Types of studies

This review will include peer reviewed publications of systematic reviews with meta-analyses or of meta-analysis of randomised clinical trials that have included a Trial Sequential Analysis and analysed at least two randomised clinical trials. A meta-analysis is a statistical approach for combining data, while a systematic review is a detailed, organised, and transparent method of gathering, appraising and synthesising data to answer a well-defined question [2]. The included studies must at least include two randomised clinical trials in at least one meta-analysis and Trial Sequential Analysis. Only studies published from January 2018 to January 2022 will be included. For practical reasons, only articles in English will be included in the study. We expect that we will identify 400 to 600 relevant publications.

#### Types of participants

We accept all participants of any race, sex, or age with any disease or condition for this review.

#### Types of interventions

We accept all types of intervention for this review.

#### Types of outcomes

All dichotomous or continuous outcomes are accepted for this review if they are analysed using both meta-analysis and Trial Sequential Analysis.

### Search strategy

The following databases will be sought:

- Medical Literature Analysis and Retrieval System Online (MEDLINE)
- The Cochrane Database of Systematic Reviews (CDSR)

Keywords used in the search strategy:

- Trial Sequential Analysis OR TSA
- Systematic Review OR Meta-analysis

The preliminary search strategy can be found in Supplemental material.

### Selection of studies

Two authors (CGR and MHO) will independently screen the title and abstract using the web-based application Covidence (www.covidence.org, Melbourne, Australia) [24]. All relevant full-text articles will be retrieved and screened for eligibility, and reasons for exclusion will be recorded. Any discrepancy will be resolved through discussion. If an agreement is not reached, a third author (CG) will resolve the disagreement. Trial selection will be shown through a flow chart following the Preferred Reporting Items for Systematic Reviews and Meta-Analyses (PRISMA) statement (Supplemental material).

### Methodological quality of the systematic reviews and meta-analyses

Two authors will independently evaluate the methodological quality in all included systematic reviews and meta-analyses using the AMSTAR 2 - Assessing the Methodological Quality of Systematic Reviews [25]. The assessment of the methodological quality of the studies will be used to evaluate whether improper use of Trial Sequential Analysis is related to other methodological flaws. Each of the 16 items will be rated, and a final rating of the overall confidence in the results of each study will be given on a scale of confidence as high, moderate, low, or critically low [25]. Any discrepancy will be resolved through discussion. If an agreement is not reached, a third author (CG) will resolve the disagreement.

### Extraction of data

Two independent authors will extract data from each included study. After extraction, all data will be compared, consensus will be reached, or a third author will be consulted to resolve disagreement.

General information (review characteristics) on each study will be extracted (author, year, medical field, intervention characteristics). For data concerning the conduct of the systematic reviews we will extract data related to the PRISMA statement on transparent reporting of systematic reviews such as comparator used, description of outcomes, number of included trials in the analyses, GRADE, methods for grading imprecision etc. [26]. For the specificities on the use and conduct of Trial Sequential Analysis we will extract data regarding the parameters used in the chosen analysis such as information on how the analysis was conducted (fixed- or random-effects, relative risk or odds ratio, etc.), and to what degree adjustments of alpha-level, and power were used. Also, details about the relative risk reduction and minimally relevant difference and how these were conceived are extracted. We will mainly report on the primary outcomes of the systematic reviews prioritising to include both dichotomous and continuous outcomes if possible. Specific data regarding the Trial Sequential Analysis will be extracted systematically through a predefined checklist made in REDCap (Research Electronic Data Capture, University of Kansas, United States) hosted at Rigshospitalet [27–29].

A prespecified extraction checklist containing items about the methodology of Trial Sequential Analysis will be prepared prior to the literature search. By using the Trial Sequential Analysis manual [30] and randomly selecting 10 systematic reviews, including meta-analysis and Trial Sequential Analysis, the most common and important steps in the analysis has been selected by the review group and synthesised into the extraction checklist. The extraction checklist contains four main categories (identification, content of the pre-registred protocol, content of the systematic review, and results), each of which has relevant questions depending on the type of outcome (dichotomous or continuous) used in the review. For more information on the extraction checklist, see Supplemental material.

### Assessment of Trial Sequential Analysis results for downgrading for imprecision

We will assess the way authors of systematic reviews or meta-analyses have used Trial Sequential Analysis for downgrading for imprecision in GRADE [16, 17] or by other methods. The GRADE approach for downgrading imprecision recommends evaluating the naïve 95% confidence interval and calculating the optimal information size [16, 31]. Thus, the approach does not emphasise the possibility to adjust alpha and beta-level, a priori decide a relative risk reduction or minimally relevant difference, and take into account heterogeneity in the analysis. We will compare the downgrading for imprecision in the included studies with the following method: imprecision in GRADE is downgraded by two levels if the accrued number of participants is below 50% of the DARIS, and one level if between 50 and 100% of DARIS. We will not expect downgrading if the cumulative Z-curve crosses the monitoring boundaries for benefit, harm, or futility, or if DARIS is reached. This method for assessing imprecision has been described and used in previous systematic reviews [32, 33]. We will examine if this methodology has an impact on how the level of imprecision is assessed and on the outcome of the systematic review or meta-analysis.

### Data analysis

The most common mistakes will be ranked according to their prevalence and comparisons of the AMSTAR 2 score in groups of studies will be made. The consequences of the most common errors and mistakes found in the literature when using Trial Sequential Analysis will be explained by examples and suggestions on how to correct these. Finally, a guideline for future reviewers will be created from the identified mistakes and errors.

Mistakes and errors will be categorised as related to the protocol, methodology, presentation of the results, and the interpretation. Each mistake and error will, furthermore, be classified as major or minor. Major mistakes or errors are those with the potential to cause a wrong conclusion. This classification will be based on a consensus by the investigators.

### Statistical considerations

The number of major or minor mistakes and errors per article will be presented as median and interquartile range. The AMSTAR 2 overall rating of confidence will be used to classify systematic reviews as high, moderate, low, or critically low confidence. Systematic reviews without any major mistakes will be compared to those with any major mistake using Wilcoxon-signed rank test or Fisher’s exact test for dichotomous outcomes.

The mistakes and errors will be presented as frequencies, with a 95% confidence interval calculated using 1-sample proportions test without continuity correction. The AMSTAR 2 score for the manuscripts where the specific mistake and error was present will be presented as a covariate. We will combine these mistakes and errors based on our recommendations, as errors referring to both protocol and presentation of results might be handled by one recommendation. Both the specified and aggregated frequency will be presented in a table related to the recommendation.

## Discussion

This review aims to assess the use of Trial Sequential Analysis in the current body of systematic reviews and meta-analyses. Trial Sequential Analysis offers important pieces of information [4, 14, 34] and provides more stringent planning of how to calculate the DARIS and interpret the imprecision in GRADE [15, 17, 18, 20, 22, 33–35] than present recommendations regarding the calculation of optimal information size and assessment of imprecision in GRADE [16]. However, the method is currently not a mandatory part of Cochrane Reviews. Arguments put forward against the use is that authors of systematic reviews and meta-analysis rarely has the power to start or stop a trial being conducted and that conclusions of meta-analyses should not be driven by statistical testing [36, 37].

Since the introduction of Trial Sequential Analysis in 2005 [19], an increasing number of authors have used the Trial Sequential Analysis to control the risk of type I and II errors and thus improve the quality of evidence and the recommendations. However, strict systematic approaches to this analysis are important as it can be misused for a ‘*cherry-picking*’ approach. Therefore, this systematic review of the methodology of current systematic reviews and meta-analyses is important for understanding and conceptualising the use of Trial Sequential Analysis. As mistakes and errors in the use of the Trial Sequential Analysis are likely to be found, these will be used to establish a guideline for future reviewers.

The strengths of this protocol are that it is pre-published and detailed prior to conducting the systematic search. As the purpose of the review is to explore the current practice of using Trial Sequential Analysis, the use of a standardised extraction template is difficult to produce as mistakes and errors comes in a variety of ways that are not always predictable. Furthermore, the extent of the work will require several persons to extract data from protocols, articles, and supplemental data from the publications. This may compromise the internal validity of the extraction. To account for this, weekly meetings are held for consensus, and all investigators are encouraged to extract in different pairs throughout the process.

Trial Sequential Analysis has been accepted as a supplementary analysis in the Cochrane Hepato-Biliary Group systematic reviews (https://hbg.cochrane.org/information-authors) and as stated in the Cochrane Handbook for Systematic Reviews of Interventions: “*…trial sequential analysis may, however, be used in the context of a prospectively planned series of randomized trials*” [38].

The results from this review will be used in the development of a comprehensive and more intuitive guideline including a standard operating procedure for conducting Trial Sequential Analysis. It is our intention that such a guideline will help future reviewers avoid these errors and mistakes. Furthermore, as the Trial Sequential Analysis software is currently being updated, the results can be incorporated in the steps when conducting the analysis to avoid mistakes.

### Stage of the review at the time of the submission

At the time of submitting this protocol, ten randomly selected systematic reviews with meta-analysis and Trial Sequential Analysis were used to test and improve the extraction checklist. Minor changes to the extraction checklist can occur during the first stage of data extraction. A preliminary search was done on the 9^th^ of July, 2021 and the final search will be conducted after the submission of this protocol.

## Supplementary information

**Additional file 1.** Supplemental information can be found in supplemental material.

## Data Availability

Not applicable.

## Abbreviations

AMSTAR 2: Assessing the methodological quality of systematic reviews 2
D^2^: Diversity
DARIS: Diversity-adjusted required information size
GRADE: Grading of Recommendations Assessment, Development and Evaluation
Pc: Proportion of participants with the outcome in the control group
RRR: Relative risk reduction
