# Supplementary material for: Major mistakes and errors in the use of Trial Sequential Analysis in systematic reviews or meta-analyses – protocol for a systematic review
Source: Syst Rev. 2022 Jun 4;11:114. doi: 10.1186/s13643-022-01987-4 (PMC9166392; doi:10.1186/s13643-022-01987-4)
Supplement: Supplementary file 1 — Additional file 1. Supplemental information can be found in supplemental material. [file 13643_2022_1987_MOESM1_ESM.pdf]

Supplemental material for

Major mistakes and Errors in the use of Trial Sequential Analysis in systematic reviews or meta-analyses – protocol for a systematic review

'METSA'

May 2022

Content

PRISMA-P 2015 Checklist 2

Preliminary search strategy 5

PRISMA flowchart 6

Extraction checklist 7

PRISMA-P 2015 Checklist

This checklist has been adapted for use with protocol submissions to *Systematic Reviews* from Table 3 in Moher D et al: Preferred reporting items for systematic review and meta-analysis protocols (PRISMA-P) 2015 statement. *Systematic Reviews* **2015** 4:**1**

Section/topic	#	Checklist item	Information reported		Line number(s)
			Yes	No	
**ADMINISTRATIVE INFORMATION**					
**Title**					
Identification	1a	Identify the report as a protocol of a systematic review	☒	☐	Page 1
Update	1b	If the protocol is for an update of a previous systematic review, identify as such	☐	☒	NA
**Registration**	2	If registered, provide the name of the registry (e.g., PROSPERO) and registration number in the Abstract	☐	☒	NA
**Authors**					
Contact	3a	Provide name, institutional affiliation, and e-mail address of all protocol authors; provide physical mailing address of corresponding author	☒	☐	Page 1
Contributions	3b	Describe contributions of protocol authors and identify the guarantor of the review	☒	☐	Page 12
**Amendments**	4	If the protocol represents an amendment of a previously completed or published protocol, identify as such and list changes; otherwise, state plan for documenting important protocol amendments	☐	☒	NA
**Support**					
Sources	5a	Indicate sources of financial or other support for the review	☒	☐	Page 12
Sponsor	5b	Provide name for the review funder and/or sponsor	☐	☒	NA
Role of sponsor/funder	5c	Describe roles of funder(s), sponsor(s), and/or institution(s), if any, in developing the protocol	☒	☐	Page 12
**INTRODUCTION**					
**Rationale**	6	Describe the rationale for the review in the context of what is already known	☒	☐	Page 3-5
**Objectives**	7	Provide an explicit statement of the question(s) the review will address with reference to participants, interventions, comparators, and outcomes (PICO)	☒	☐	Page 5
**METHODS**					
**Eligibility criteria**	8	Specify the study characteristics (e.g., PICO, study design, setting, time frame) and report characteristics (e.g., years considered,	☒	☐	Page 6

Section/topic	#	Checklist item	Information reported		Line number(s)
			Yes	No	
		language, publication status) to be used as criteria for eligibility for the review			
**Information sources**	9	Describe all intended information sources (e.g., electronic databases, contact with study authors, trial registers, or other grey literature sources) with planned dates of coverage	☒	☐	Page 7
**Search strategy**	10	Present draft of search strategy to be used for at least one electronic database, including planned limits, such that it could be repeated	☒	☐	Supplemental material
STUDY RECORDS					
Data management	11a	Describe the mechanism(s) that will be used to manage records and data throughout the review	☒	☐	Page 7
Selection process	11b	State the process that will be used for selecting studies (e.g., two independent reviewers) through each phase of the review (i.e., screening, eligibility, and inclusion in meta-analysis)	☒	☐	Page 7
Data collection process	11c	Describe planned method of extracting data from reports (e.g., piloting forms, done independently, in duplicate), any processes for obtaining and confirming data from investigators	☒	☐	Page 8
**Data items**	12	List and define all variables for which data will be sought (e.g., PICO items, funding sources), any pre-planned data assumptions and simplifications	☒	☐	Page 8
**Outcomes and prioritization**	13	List and define all outcomes for which data will be sought, including prioritization of main and additional outcomes, with rationale	☐	☒	NA
**Risk of bias in individual studies**	14	Describe anticipated methods for assessing risk of bias of individual studies, including whether this will be done at the outcome or study level, or both; state how this information will be used in data synthesis	☒	☐	Page 7-8
DATA					
**Synthesis**	15a	Describe criteria under which study data will be quantitatively synthesied	☒	☐	Page 9-10
	15b	If data are appropriate for quantitative synthesis, describe planned summary measures, methods of handling data, and methods of combining data from studies, including any planned exploration of consistency (e.g., I^2, Kendall's tau)	☐	☒	NA
	15c	Describe any proposed additional analyses (e.g., sensitivity or subgroup analyses, meta-regression)	☐	☒	NA

Section/topic	#	Checklist item	Information reported		Line number(s)
			Yes	No	
	15d	If quantitative synthesis is not appropriate, describe the type of summary planned	☒	☐	Page 9-10
**Meta-bias(es)**	16	Specify any planned assessment of meta-bias(es) (e.g., publication bias across studies, selective reporting within studies)	☐	☒	NA
**Confidence in cumulative evidence**	17	Describe how the strength of the body of evidence will be assessed (e.g., GRADE)	☐	☒	NA

Preliminary search strategy

Cochrane Database of Systematic Reviews
#1 ("trial sequential" and (analys* or (monitoring next boundar*))) or (cumulative next meta-analys*)

MEDLINE Ovid (2004 to July 2021) (1761 hits)
1. ((trial sequential and (analys* or monitoring boundar*)) or cumulative meta-analys*).mp. [mp=title, abstract, original title, name of substance word, subject heading word, floating sub-heading word, keyword heading word, organism supplementary concept word, protocol supplementary concept word, rare disease supplementary concept word, unique identifier, synonyms]

2. limit 1 to yr="2004 -Current"

PRISMA flowchart

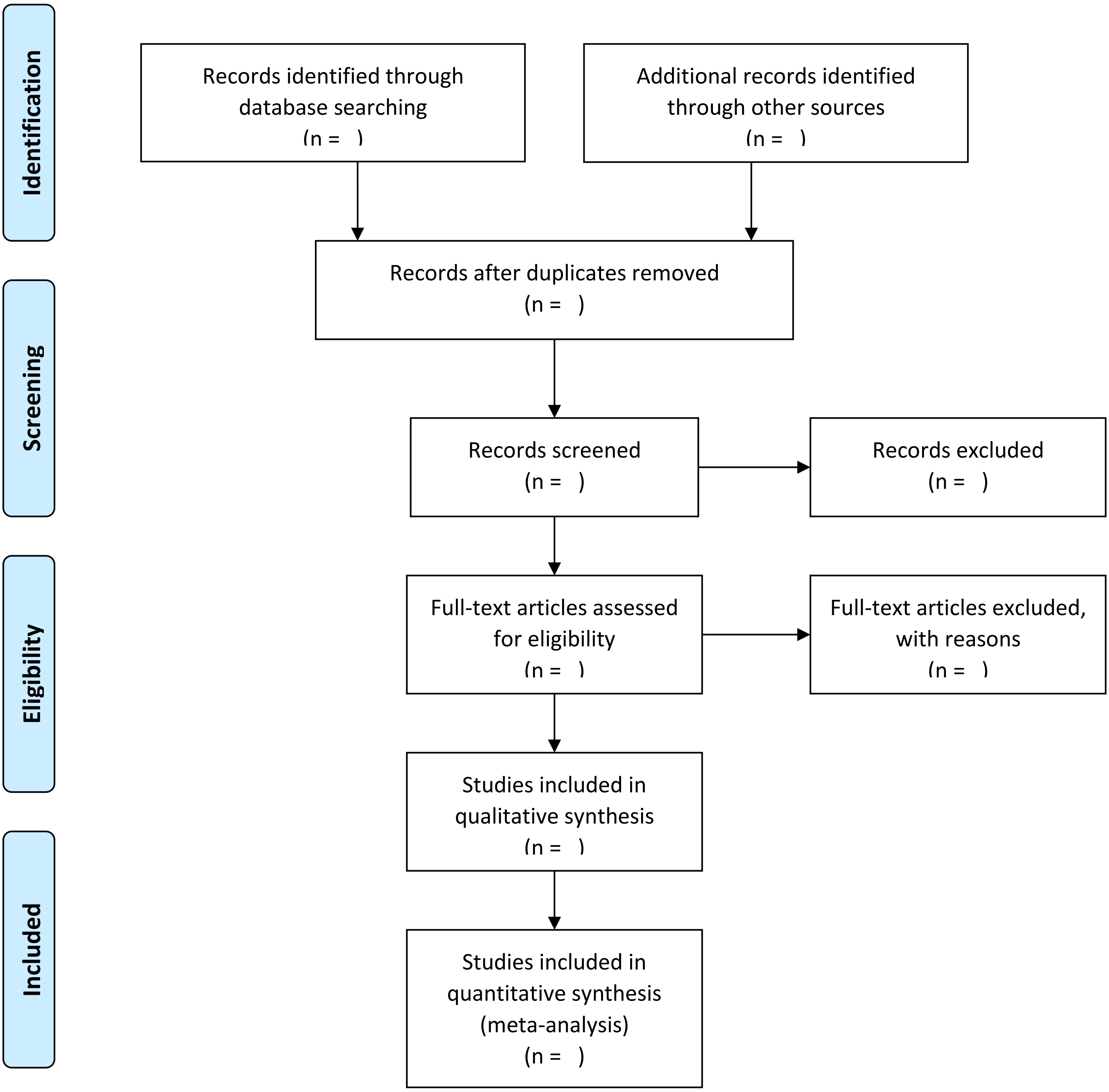

From: Moher D, Liberati A, Tetzlaff J, Altman DG, The PRISMA Group (2009). *P*referred *R*eporting *I*tems for *S*ystematic Reviews and *M*eta-*A*nalyses: The PRISMA statement. PLoS Med 6(7): e1000097. doi:10.1371/journal.pmed1000097

Extraction checklist

1. ID

Record ID

____

Standard procedure for data extraction

General workflow Source files via Covidence: Major mistakes and Errors conducted using Trial Sequential Analysis in systematic reviews or meta-analyses (METSA) (covidence.org) Filter by #CovidenceID Add missing files to covidence (e.g. protocol or supplementary material)   Go through REDcap instruments 1-5 in the pre-specified order

Handling missing data or incompatibility When a numerical value is required but none is available, input: "-99" When text is required but none is available, input: "NA" If question is missing appropriate answer, consult JBM

Misc. When citing paper, place citation in " " Records are added using the attached list If in doubt about any of the above, consult CGR or JBM

[Attachment: "metsa_extraction list.pdf"]

Reviewer identification

- ◯ CGR
- ◯ EBP
- ◯ JBM
- ◯ JHPR
- ◯ JHS
- ◯ MHO
- ◯ SHH
- ◯ Ej tildelt

REDcap extraction number

____

CovidenceID #

____

DOI-number of the systematic review

____
(Copy/paste the record number)

Provide first author surname and publication year

____
(e.g. Johnson 2021)

In which country did the corresponding author have it's address/primary affiliation?

- ◯ Afghanistan
- ◯ Albania
- ◯ Algeria
- ◯ Andorra
- ◯ Angola
- ◯ Antigua & Deps
- ◯ Argentina
- ◯ Armenia
- ◯ Australia
- ◯ Austria
- ◯ Azerbaijan
- ◯ Bahamas
- ◯ Bahrain
- ◯ Bangladesh
- ◯ Barbados
- ◯ Belarus
- ◯ Belgium
- ◯ Belize
- ◯ Benin
- ◯ Bhutan
- ◯ Bolivia
- ◯ Bosnia Herzegovina
- ◯ Botswana
- ◯ Brazil
- ◯ Brunei
- ◯ Bulgaria
- ◯ Burkina
- ◯ Burundi
- ◯ Cambodia
- ◯ Cameroon
- ◯ Canada
- ◯ Cape Verde
- ◯ Central African Rep
- ◯ Chad
- ◯ Chile
- ◯ China
- ◯ Colombia
- ◯ Comoros
- ◯ Congo
- ◯ Congo (Democratic Rep)
- ◯ Costa Rica
- ◯ Croatia
- ◯ Cuba
- ◯ Cyprus
- ◯ Czech Republic
- ◯ Denmark
- ◯ Djibouti
- ◯ Dominica
- ◯ Dominican Republic
- ◯ East Timor
- ◯ Ecuador
- ◯ Egypt
- ◯ El Salvador
- ◯ Equatorial Guinea
- ◯ Eritrea
- ◯ Estonia
- ◯ Ethiopia
- ◯ Fiji
- ◯ Finland
- ◯ France
- ◯ Gabon
- ◯ Gambia
- ◯ Georgia
- ◯ Germany
- ◯ Ghana
- ◯ Greece
- ◯ Grenada
- ◯ Guatemala
- ◯ Guinea

◯ Guinea-Bissau
◯ Guyana
◯ Haiti
◯ Honduras
◯ Hungary
◯ Iceland
◯ India
◯ Indonesia
◯ Iran
◯ Iraq
◯ Ireland {Republic}
◯ Israel
◯ Italy
◯ Ivory Coast
◯ Jamaica
◯ Japan
◯ Jordan
◯ Kazakhstan
◯ Kenya
◯ Kiribati
◯ Korea North
◯ Korea South
◯ Kosovo
◯ Kuwait
◯ Kyrgyzstan
◯ Laos
◯ Latvia
◯ Lebanon
◯ Lesotho
◯ Liberia
◯ Libya
◯ Liechtenstein
◯ Lithuania
◯ Luxembourg
◯ Macedonia
◯ Madagascar
◯ Malawi
◯ Malaysia
◯ Maldives
◯ Mali
◯ Malta
◯ Marshall Islands
◯ Mauritania
◯ Mauritius
◯ Mexico
◯ Micronesia
◯ Moldova
◯ Monaco
◯ Mongolia
◯ Montenegro
◯ Morocco
◯ Mozambique
◯ {Burma}
◯ Namibia
◯ Nauru
◯ Nepal
◯ Netherlands
◯ New Zealand
◯ Nicaragua
◯ Niger
◯ Nigeria
◯ Norway
◯ Oman
◯ Pakistan
◯ Palau
◯ Panama
◯ Papua New Guinea
◯ Paraguay
◯ Peru
◯ Philippines
◯ Poland

◯ Portugal
◯ Qatar
◯ Romania
◯ Russian Federation
◯ Rwanda
◯ St Kitts & Nevis
◯ St Lucia
◯ Saint Vincent & the Grenadines
◯ Samoa
◯ San Marino
◯ Sao Tome & Principe
◯ Saudi Arabia
◯ Senegal
◯ Serbia
◯ Seychelles
◯ Sierra Leone
◯ Singapore
◯ Slovakia
◯ Slovenia
◯ Solomon Islands
◯ Somalia
◯ South Africa
◯ South Sudan
◯ Spain
◯ Sri Lanka
◯ Sudan
◯ Suriname
◯ Swaziland
◯ Sweden
◯ Switzerland
◯ Syria
◯ Taiwan
◯ Tajikistan
◯ Tanzania
◯ Thailand
◯ Togo
◯ Tonga
◯ Trinidad & Tobago
◯ Tunisia
◯ Turkey
◯ Turkmenistan
◯ Tuvalu
◯ Uganda
◯ Ukraine
◯ United Arab Emirates
◯ United Kingdom
◯ United States
◯ Uruguay
◯ Uzbekistan
◯ Vanuatu
◯ Vatican City
◯ Venezuela
◯ Vietnam
◯ Yemen
◯ Zambia
◯ Zimbabwe

CTU affiliated?

◯ Yes
◯ No

Which files were evaluated for this entry?	☐ Main paper<br>☐ Supplementary materials<br>☐ Prepublished protocol<br>☐ Pretrial registration (PROSPERO or elsewhere)<br>☐ Other<br>(Include also material, that didn't contain relevant information, e.g., supplementary material when not relevant for TSA)
Please describe which files were evaluated	__________________________________
Study details	
Choose the appropriate study type	◯ Systematic review (with pre-published/pre-registred protocol)<br>◯ Meta-analysis (without pre-published/pre-registred protocol)<br>(Systematic reviews aim to minimize bias through the use of pre-specified research questions and methods that are documented in protocols)
Was the review an update of a previous systematic review?	◯ Yes<br>◯ No
When the review is an update of an existing review, please identify the protocol for updated review. If no protocol has been made specifically for the updated review, assess whether the updated review explicitly and firmly claims to adhere to methods defined in previous protocols/review versions. Otherwise, mark the updated review as having no predefined protocol.	
Please describe the review history (e.g. list previous versions, protocols, and describe if the update was performed by a different author group)	__________________________________
Has the protocol been made public before data extraction began?	◯ Yes<br>◯ No<br>◯ Unclear
Was this a Cochrane Review?	◯ Yes<br>◯ No
Medical field and intervention type	
What was the overall research question?	__________________________________

What is the medical field(s) of interest in the article?

- ☐ Allergy and immunology
- ☐ Alternative medicine
- ☐ Anesthesiology
- ☐ Cardiology
- ☐ Clinical biochemistry
- ☐ Dentistry
- ☐ Dermatology
- ☐ Diagnostic radiology
- ☐ Emergency medicine
- ☐ Endocrinology
- ☐ Family medicine
- ☐ Gastroenterology
- ☐ Gastroentestinal surgery
- ☐ Genetics
- ☐ Geriatric medicine
- ☐ Gynecology
- ☐ Hematology
- ☐ Hepatology
- ☐ Infectious diseases
- ☐ Intensive care
- ☐ Internal medicine
- ☐ Medical genetics
- ☐ Nephrology
- ☐ Neurology
- ☐ Neurosurgery
- ☐ Nuclear medicine
- ☐ Obstetrics
- ☐ Oncology
- ☐ Ophthalmology
- ☐ Orthopedic surgery
- ☐ Oto-rhino-laryngology (ear-nose-throat)
- ☐ Palliative care
- ☐ Pathology
- ☐ Pediatrics
- ☐ Pediatric psychiatry
- ☐ Physical medicine and rehabilitation
- ☐ Plastic surgery
- ☐ Preventive medicine
- ☐ Pulmonary diseases
- ☐ Psychiatry
- ☐ Thoracic surgery
- ☐ Urology
- ☐ Vascular surgery
- ☐ Other

Please describe the medical field of interest in the article

____

What was the overall goal(s) with the intervention?

- ☐ Cure from disease
- ☐ Prevention of disease
- ☐ Prevention of complication to treatment (incl. perioperative care)
- ☐ Disease control
- ☐ Rehabilitation
- ☐ Palliation (pain management)
- ☐ Diagnostic
- ☐ Life support
- ☐ Other

Please describe the goal of the intervention

____

Which intervention type(s) was examined in the article?

- ☐ Pharmacological
- ☐ Surgical
- ☐ Psychotherapy
- ☐ Diagnostic
- ☐ Medical device
- ☐ Nutritional
- ☐ Exercise/rehabilitation
- ☐ Lifestyle
- ☐ Alternative
- ☐ Other

Which intervention was examined in the article?

____

To which specific diagnosis/health issue was the intervention applied?

____

3. Study description (non-repeatable)

Record ID

____

Instrument 3 contents

Study details
Outcomes TSA documentation
Study summary and conclusions
GRADE Misc. comments

To whom were the intervention group(s) compared (what is the type of comparator)?

- ☐ Standard care
- ☐ Placebo
- ☐ Healthy controls
- ☐ No treatment
- ☐ Other

Please describe the comparator

____

Was the literature search date defined?

- ◯ Yes
- ◯ No

Date of literature search (final date)

____
(If authors state month only, e.g, December 2019, choose the first day of the month, i.e., 01-12-2019)

Outcomes

Authors classify outcome measures inconsistently. In this database, outcomes are classified by the following definitions:

Primary or "main" outcome(s): The central research question(s) of the study and the reason that the study is done

Secondary or "additional" outcome(s): Research question(s) applied to all participants of the included studies. Provides additional information on the intervention effect.

Exploratory outcome(s): Research question(s) applied to all participants of the included studies. Primarily used for hypothesis generation.

Subgroup analysis: analyses of any of the outcomes described above, but in a subset of the sample population or the included trials. Can provide information on sources of heterogeneity (variations in demographics, intervention, etc.)
(Cochrane definition: https://handbook-5-1.cochrane.org/chapter_9/9_6_2_what_are_subgroup_analyses.htm)

How many primary outcomes were investigated in this study?

____

List all primary outcomes in study

____
(Place each outcome on a separate line)

How were the primary outcomes labelled by the authors?	◯ "Primary"<br>◯ "Main"<br>◯ Not labelled<br>◯ Other
Please describe how the primary outcomes were labelled by the authors	__________________________________
How many secondary outcomes were investigated in this study?	____________________________
List all secondary outcomes in study	__________________________________<br>(Place each outcome on a separate line)
How were the secondary outcomes labelled by the authors?	◯ "Secondary"<br>◯ "Additional"<br>◯ "Subgroup analysis"<br>◯ Not labelled<br>◯ Other
Please describe how the secondary outcomes were labelled by the authors	__________________________________
How many exploratory outcomes were investigated in this study?	____________________________
List all exploratory outcomes in study	__________________________________<br>(Place each outcome on a separate line)
How were the exploratory outcomes labelled by the authors?	◯ "Exploratory"<br>◯ "Additional"<br>◯ "Observational"<br>◯ "Subgroup analysis"<br>◯ Not labelled<br>◯ Other
Please describe how the exploratory outcomes were labelled by the authors	__________________________________
Were any subgroup analysis performed?	◯ Yes<br>◯ No
Please describe the subgroup analysis performed in the study	__________________________________<br>(Describe to which outcome(s) the subgroup analysis was performed)

How were the subgroup analyses labelled by the authors?	◯ "Subgroup analysis"<br>◯ "Secondary"<br>◯ "Exploratory"<br>◯ "Additional"<br>◯ "Observational"<br>◯ Not labelled<br>◯ Other
Please describe how the subgroup authors were labelled by the authors	__________________________________
To which outcome(s) and or subgroup analyses was TSA applied?<br><br>Authors may choose to apply TSA but not share the graph and only report TSA CI's - look closely!	__________________________________<br>(Include also outcomes to which TSA was planned, but data was missing or sample size was too low to perform the TSA)
If applicable, please list outcomes to which TSA was planned, but could not be performed due to lack of data	__________________________________
TSA - Documentation	
Has the authors stated which version of the Trial Sequential Analysis software they used?	◯ Yes<br>◯ No
Was the Trial Sequential Analysis software correctly referenced?	◯ Yes<br>◯ No<br>◯ Not referenced<br>(Minimum requirement: exact software version e.g. 0.9.5.10 + source e.g. ctu.dk)
Reference	__________________________________<br>(Please provide information. If "No" state which references were actually provided or "no references provided".)
Was the choice of statistical methods for Trial Sequential Analysis referenced?	◯ Yes<br>◯ No<br>(Either the manual or relevant references should be cited)

Which reference(s) was used?	☐ Brok J et al. Trial sequential analysis reveals insufficient information size and potentially false positive results in many metaanalyses. J. Clin. Epidemiol.2008;61 (8), 763-769.<br>☐ Brok et al. Apparently conclusive meta-analyses may be inconclusive: trial sequential analysis adjustment of random error risk due to repetitive testing of accumulating data in apparently conclusive neonatal meta-analyses. Int J Epidemiol. 2009;38(1):287-298<br>☐ Imberger G et al. False-positive findings in Cochrane meta-analyses with and without application of trial sequential analysis: an empirical review. British Medical Journal Open 2016; 6: e011890<br>☐ Jakobsen JC et al. Thresholds for statistical and clinical significance in systematic reviews with meta-analytic methods. BMC Med Res Methodol 2014;14:120.<br>☐ Thorlund K et al. Can trial sequential monitoring boundaries reduce spurious inferences from meta-analyses? Int J Epidemiol 2009;38:276-86.<br>☐ Thorlund K et al. User manual for Trial Sequential Analysis (TSA). Available: www.ctu.dk/tsa/files/tsa_manual.pdf<br>☐ Wetterslev J et al. Trial sequential analysis may establish when firm evidence is reached in cumulative meta-analysis. J. Clin. Epidemiol. 2008;61 (1), 64-75.<br>☐ Wetterslev J et al. Estimating required information size by quantifying diversity in random-effects model meta-analyses. BMC Med Res Methodol. 2009;9:86<br>☐ Wetterslev J et al. Trial sequential analysis in systematic reviews with meta-analysis. BMC Med Res Methodol 2017;17:39.<br>☐ Other
Other references	__________________________________<br>(Please insert complete reference - If the reference is relevant inform Christian Riberholt by e-mail)
Did the authors provide a TSA-report (or other formative report)?	◯ Yes<br>◯ No<br>(Check supplementary materials)
Study summary and conclusions	
How many RCT's were included in the article?	__________________________________<br>(Please list the actual number of RCT included.)

How many RCT's were included in the largest meta analysis?

____
(Largest by sample size. If the RCT has multiple groups and, therefore, is included in the meta-analysis more than once, count each data entry and make a note in the 'general comment field' below.)

How many non-randomized trials were included in the article?

____

How many non-randomized trials were included in the largest meta analysis?

____
(Largest by sample size)

Was the meta analysis presented in a forest plot?

◯ Yes
◯ No

The following questions ask for the FINAL conclusions of the study and not the "subconclusions" of the individual analyses, i.e., the results of individual TSA analyses or forest plots

What did the authors conclude about the intervention effect on the outcome(s) analyzed with TSA?

Outcome(s) analyzed with TSA:
[outc_tsa]

◯ Inconclusive
◯ Beneficial
◯ Harmful
◯ Futile
◯ Other/multiple
(Reminder to read the entire conclusion - authors may conclude that data indicates effect, only to conclude that the data quality was too low and thus the final conclusion is "inconclusive")

Please describe the conclusion (or copy/paste)

____

Were there any conclusions on the waste of research (relevant when limits for benefit, futility or harm are reached) ?

◯ Yes
◯ No

What did the authors conclude about the waste of research?

____

GRADE

Was GRADE used for one or more outcomes to which TSA was applied?

(including outcomes where TSA wasn't performed due to lack of data)

◯ Yes, one
◯ Yes, more than one
◯ No
(GRADE = Grading of Recommendations, Assessment, Development and Evaluations)

How many outcomes were GRADE'd AND analyzed with TSA?

(including outcomes where TSA wasn't performed due to lack of data)

____

Describe to which outcome(s) GRADE was applied

____
(Describe only outcomes to which TSA was applied)

Did the Trial Sequential Analysis explicitly influence the GRADE assessment?

◯ Yes ◯ No

Describe how Trial Sequential Analysis influenced GRADE evaluation

____

How was the outcome GRADE'd?

◯ High
◯ Moderate
◯ Low
◯ Very low

How were the outcomes GRADE'd?

☐ High
☐ Moderate
☐ Low
☐ Very low

Were the downgrade methods for imprecision described?

◯ Yes
◯ No

Which parameters influenced the grading of imprecision in GRADE?

☐ Imprecision downgraded 2 levels if DARIS < 50%; 1 level if DARIS between 50-100%; 0 levels if DARIS is reached 100% or if the cumulative Z-curve crosses boundaries for futility, benefit or harm
☐ Imprecision downgraded but the cut-offs were different than (1) or not specified (describe below)
☐ Imprecision was evaluated using the Trial Sequential adjusted confidence interval
☐ Judgement of the width of conventional 95% CI's
☐ Other

Describe how

____

How much was imprecision downgraded?

◯ 1 level
◯ 2 levels
◯ Imprecision was not explicitly downgraded
◯ Other

How much was imprecision downgraded?

☐ 1 level
☐ 2 levels
☐ Imprecision was not explicitly downgraded
☐ Other

Please describe how imprecision was downgraded

____

Misc.

General comments on study issues or other noteworthy details not described elsewhere:	______________________________ (Please fill in general comments that has not been elucidated in the questionaire)
Did the article mention/apply Bayesian approaches to statistical analysis?<br><br>(search article for "bayes")	◯ Yes<br>◯ No<br>(This question is used to screen for Bayesian content for a potential future project)

4. TSA Description (repeatable)

Record ID

____

Instrument 4 contents

TSA - Outcome details TSA - Methods Dichotomous/continuous TSA - Graphical presentation and results Graphics Results

TSA - Outcome details

How many outcomes were analyzed with TSA?

____
(Those that were actually analyzed, i.e., not those with insufficient data - including subgroup analyses)

Please list all outcomes and subgroups analyzed with TSA (only in first instance of "Instrument 4 - TSA description)

____

In the previous instrument, you listed the following:
[outc_tsa]

And noted that TSA wasn't performed on the following outcomes due to lack of data:
[outc_tsa_miss]

REMINDER! When multiple outcomes have been analyzed with TSA, repeat the TSA instrument and extract data from one dichotomous and one continuous outcome measure if applicable.

Dichotomous outcomes
a - Identify TSAs performed on dichotomous outcome measures (if NA, skip and go to 2a)
b - if a>1, identify TSAs from highest rank (primary>secondary>explorative>subgroup)
c - if b>1, identify TSA with highest acquired information size (AIS)
d - if c>1, identify TSA with highest AIS/RIS
e - if d>1, pick the one that appears in the article first Continuous outcomes
a - Identify TSAs performed on continuous outcome measures (if NA, skip)
b - if a>1, identify TSAs from highest rank (primary>secondary>explorative>subgroup)
c - if b>1, identify TSA with highest acquired information size (AIS)
d - if c>1, identify TSA with highest AIS/RIS
e - if d>1, pick the one that appears in the article first

What is the outcome to which TSA was applied?

____
(Please copy/paste from paper)

What is the data type for the outcome?

- ◯ Dichotomous
- ◯ Continuous

What is the type of outcome?

- ◯ Primary
- ◯ Secondary
- ◯ Exploratory
- ◯ Subgroup analysis

Was the outcome analyzed with TSA analyzed in a network meta analysis (NMA)?

- ◯ Yes
- ◯ No

Which analysis model was used for the outcome analyzed with TSA?	◯ Fixed-effect model<br>◯ Random-effects models<br>◯ Fixed- and random-effects models<br>◯ Other<br>◯ None
Please provide additional model details and/or describe other models	__________________________________
TSA - Methods	
This instrument asks for the details of the TSA analysis. Often, only one alpha-level will be chosen for all analyses (TSA, forest plots, etc.) but in the case that alpha-levels (or other details) differ, please note the value used for the TSA analysis and describe the difference in comments at the end of the instrument.<br><br>Values for each TSA should be presented in the figure, above the figure (figure head) or below the figure (legend), but will often be found in the description of statistical methods and/or results section.	
Was the alpha-level defined?	◯ Yes<br>◯ Yes, conventional at 0.05<br>◯ No<br>(Provide values under comments)
Provide the alpha-level defined	___________________________<br>(Please provide as number, not percentage (e.g., 0.05 and not 5))
Describe the choice of alpha, if possible	__________________________________<br>((if alpha=0.05, note: "conventional"))
Was the alpha level explicitly corrected for multiplicity?	◯ Yes<br>◯ No
Which method was used to adjust alpha for multiplicity?	☐ Bonferroni<br>☐ Other
Please describe the method used for multiplicity correction	__________________________________
Was the power defined?	◯ Yes<br>◯ Yes, conventional at 0.8<br>◯ Yes, conventional at 0.9<br>◯ No
Provide the power defined	___________________________<br>(Please provide as number, not percentage (e.g., 0.8 and not 80))
Describe the choice of power used in the Trial Sequential Analysis	__________________________________<br>((if power=0.8 or 0.9, note: "conventional"))

Was heterogeneity correction used for Trial Sequential Analysis?	◯ Inconsistency (I-square)<br>◯ Diversity (D-square)<br>◯ Not described, but no heterogeneity found in meta analysis (I^2=0%)<br>◯ Not described / not clear<br>◯ Other<br>◯ No
Describe the choice of heterogeneity used in the Trial Sequential Analysis	__________________________________
Was the value for heterogeneity correction provided?	◯ Yes<br>◯ No
Please provide values for heterogeneity correction	___________________________
TSA methods - dichotomous outcome	
Which association measure was used for analysis in the dichotomous outcome?	☐ Relative risk (RR)<br>☐ Risk difference (RD)<br>☐ Odds Ratio (OR)<br>☐ Peto OR<br>☐ Other
Please provide information on association measures	__________________________________
Was it relevant to correct for zero events in one group?<br>(i.e. did one group have zero events?)	◯ Yes<br>◯ No<br>◯ Unclear (not described)
How did they correct for zero events?	☐ Constant<br>☐ Reciprocal<br>☐ Empirical<br>☐ Correction value = 1<br>☐ Correction value = 0.5<br>☐ Correction value = 0.01<br>☐ Correction value = 0.001<br>☐ Trials with no events were excluded<br>☐ No correction described<br>☐ Other methods<br>(Check all relevant boxes)
Please describe the method used for correction of zero events	__________________________________
Was proportion of events in the control group (Pc) defined?	◯ Yes, with value<br>◯ Yes, but value not reported<br>◯ No<br>(Provide values under comments)

Where did the choice for the proportion of events in the control group (Pc) come from?	☐ From other published meta-analysis<br>☐ From one or more published randomised trials<br>☐ From one or more published observational studies<br>☐ From the observed data in their meta-analysis<br>☐ Unclear<br>☐ Other<br>☐ Not mentioned
Please describe the choice of Pc	__________________________________
Please provide the value defined for Pc	____________________________
Was the relative risk reduction (RRR) defined?	◯ Yes<br>◯ No
Please provide the RRR defined	____________________________<br>(Input as decimal number, not percentage)
Was it described how the relative risk reduction (RRR) was defined?	☐ From a published meta-analysis<br>☐ From one or more published randomised trials<br>☐ From one or more observational studies<br>☐ From the observed data in their meta-analysis<br>☐ Clinical experience (pick this if authors have assessed the RRR as "realistic" or similar)<br>☐ Other<br>☐ No
Describe the choice of RRR used in the Trial Sequential Analysis	__________________________________
TSA methods - continuous outcome	
Which association measure was used in the analysis for the continuous outcome?	☐ Mean difference (MD)<br>☐ Standardized mean difference (SMD)<br>☐ Other<br>(Weighted mean difference (WMD) is the same as MD)
Please provide information on association measures	__________________________________
Was variance defined?	◯ Yes<br>◯ No
Provide the variance defined	____________________________
Describe the choice of variance used in the Trial Sequential Analysis	__________________________________

Was MIREDIF defined?	◯ Yes<br>◯ No<br>("MIREDIF" = Minimal relevant difference. Provide values under comments)
Provide the MIREDIF defined	__________________________________
Describe the choice of MIREDIF used in the Trial Sequential Analysis	__________________________________
TSA - Graphical presentation and results	
Graphics	
Was the Trial Sequential Analysis presented in a figure?	◯ Yes<br>◯ No
Were was the TSA graph presented?	◯ In article<br>◯ In supplementary materials<br>◯ Other
Please describe where the TSA graph was presented	__________________________________
Are the conventional 5% limits correctly outlined?	◯ Yes<br>◯ No<br>◯ Not outlined
Why was it not correct?	__________________________________
Was the area of benefit above or below the x-axis?	◯ Above<br>◯ Below
Is the monitoring boundary of benefit correctly outlined?	◯ Yes<br>◯ No<br>◯ No, not outlined due to software limitation if first trial exceeds RIS/DARIS<br>◯ No, not outlined (but not relevant with only negative Z-scores)
Why was it not correct?	__________________________________
Is the monitoring boundary of harm correctly outlined?	◯ Yes<br>◯ No<br>◯ No, not outlined due to software limitation if first trial exceeds RIS/DARIS<br>◯ No, not outlined (but not relevant with only positive Z-scores)

Why was it not correct?

____

Is the area of futility (the inner wedge) correctly outlined?

- ◯ Yes
- ◯ No
- ◯ No, not outlined due to software limitation if first trial exceeds RIS/DARIS or cumulative sample size doesn't reach area of futility
- ◯ Inner wedge not applied

Why was it not correct?

____

Is the line indicating required information size (RIS or DARIS) correctly outlined?

- ◯ Yes
- ◯ No
- ◯ No, not outlined due to software limitation if first trial exceeds RIS/DARIS

Why was it not correct?

____

Is the cumulative Z-curve correctly outlined?

- ◯ Yes
- ◯ No
- ◯ No (trial spacing equal and not based on trial sizes)
- ◯ Not outlined

Why was it not correct?

____

Was the graphical presentation of the TSA different or flawed in a way not already described?

- ◯ Yes
- ◯ No

Please describe flaws and/or differences

____

Results

How many trials were analyzed?

____
(If discrepancy between figure and text/table/forest plot - report from figure! If trials are included more than once due to multiple groups, count each data entry and make a note in the 'Misc. comments on TSA' at the end.)

What was the acquired information size?

____
(If NA, input '-99')

Was the AIS directly extracted from figure or figure legend or was it calculated by the extractor, e.g., from forest plots?	◯ Directly extracted (e.g., from figure, figure legend or text)<br>◯ Calculated (e.g., summing sample sizes from forest plot)
Did the authors present a required information size for the analysis?	☐ Yes, diversity-adjusted required information size (DARIS)<br>☐ Yes, non-adjusted required information size (RIS)<br>☐ Yes, but not clear if RIS or DARIS<br>☐ No
What was the estimated non-adjusted or diversity adjusted required information size (RIS or DARIS)?	__________________________________
Was the non-adjusted or diversity adjusted required information size (RIS or DARIS) estimated from number of events or population size?	◯ Number of events<br>◯ Population size<br>◯ Not available

If futility wedge is missing, area 6 is limited to the area past RIS/DARIS

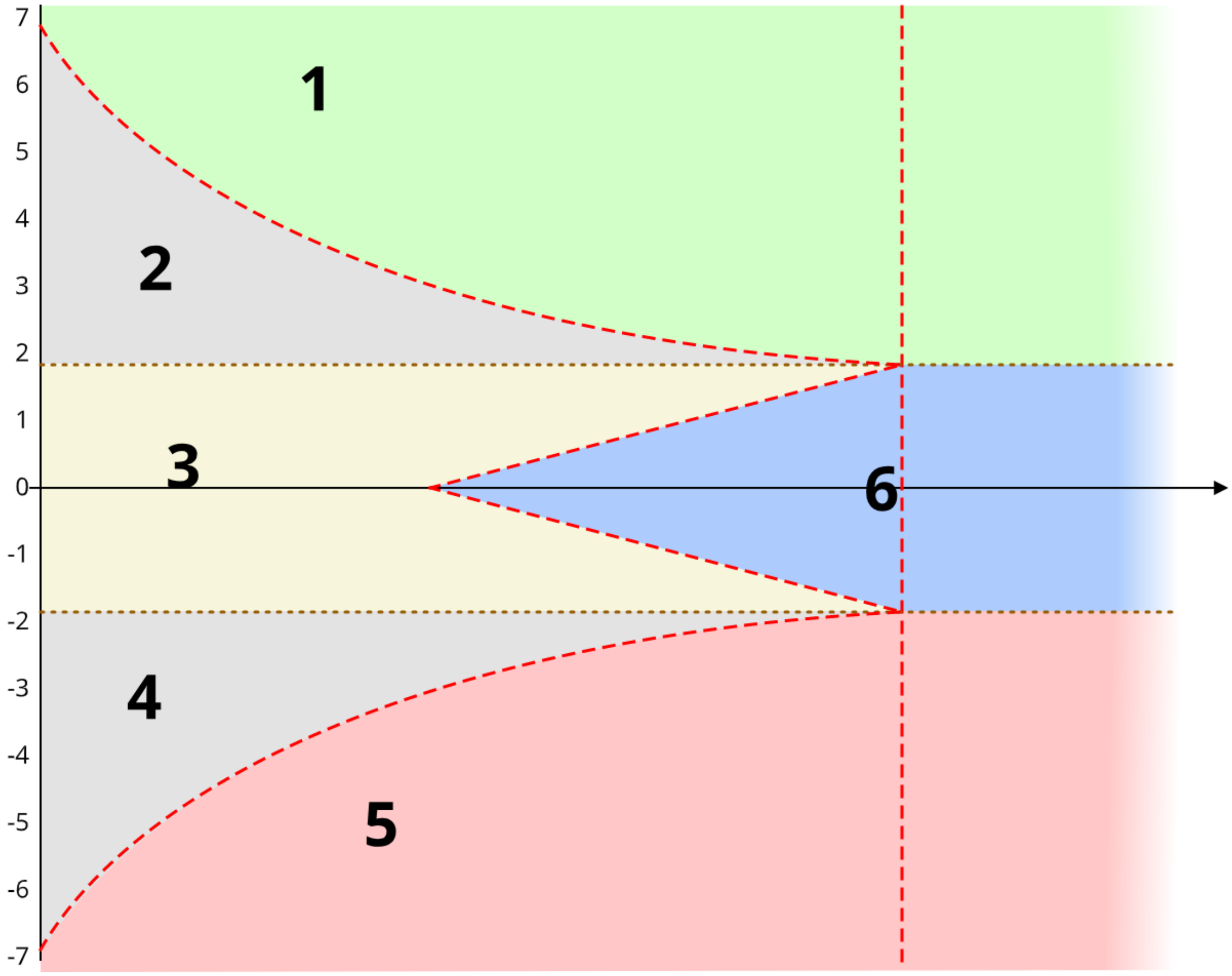

Please note in the table below were the individual trials of the Z-curve was placed in the TSA graphic (see area description in image above) - if TSA contains more than 84 studies, please leave a comment using the comment function and contact JBM

Trial number TSA graph area (input integer: 1-6)	Trial number TSA graph area (input integer: 1-6)	Trial number TSA graph area (input integer: 1-6)
1 ______	2 ______	3 ______
4 ______	5 ______	6 ______
7 ______	8 ______	9 ______
10 ______	11 ______	12 ______
13 ______	14 ______	15 ______
16 ______	17 ______	18 ______
19 ______	20 ______	21 ______
22 ______	23 ______	24 ______
25 ______	26 ______	27 ______
28 ______	29 ______	30 ______
31 ______	32 ______	33 ______
34 ______	35 ______	36 ______
37 ______	38 ______	39 ______
40 ______	41 ______	42 ______
43 ______	44 ______	45 ______
46 ______	47 ______	48 ______
49 ______	50 ______	51 ______
52 ______	53 ______	54 ______
55 ______	56 ______	57 ______
58 ______	59 ______	60 ______
61 ______	62 ______	63 ______
64 ______	65 ______	66 ______
67 ______	68 ______	69 ______
70 ______	71 ______	72 ______
73 ______	74 ______	75 ______
76 ______	77 ______	78 ______
79 ______	80 ______	81 ______
82 ______	83 ______	84 ______

Did the authors provide Trial Sequential adjusted confidence intervals?

◯ Yes
◯ Yes, but mislabelled
◯ No
(Should be labelled as Trial Sequential adjusted confidence interval or TSA CI (not 95%CI))

In what way did they mislabel the Trial Sequential adjusted confidence interval?

____

What was the estimated effect size for the outcome analyzed with TSA?

Point estimate (e.g., mean dif, RR)

______

Conventional CI, lower limit

______

Conventional CI, upper limit

______

TSA adjusted CI, lower limit

______

TSA adjusted CI, upper limit

______

Misc. comments on TSA not considered elsewhere

____

Please rate the overall transparency of the TSA analysis<br><br>You may consider the following variables:<br><br>Discrepancies between graph and text or between text segments Graphic quality of figure - including labels Specification of included trials Acquired information size Required information size (RIS or DARIS) Relative risk reduction or MIREDIF Heterogeneity Proportion of events in the control population (only dichotomous outcomes) Variance (only continuous outcomes) Chosen association measure	◯ Excellent / really good (all parameters transparently presented)<br>◯ Good (few parameters missing)<br>◯ Poor (several important parameters missing)<br>◯ Useless (graph unreadable and/or crucial parameters missing)
Which parameters caused you to rate the overall transparency as "[tsares_trans]" ?	☐ Discrepancies between graph and text or between text segments<br>☐ Graphic quality of figure - including labels<br>☐ Specification of included trials<br>☐ Acquired information size<br>☐ Required information size (RIS or DARIS)<br>☐ Relative risk reduction or MIREDIF<br>☐ Heterogeneity<br>☐ Proportion of events in the control population (only dichotomous outcomes)<br>☐ Variance (only continuous outcomes)<br>☐ Chosen association measure<br>☐ Other
Please describe which other parameters influenced your rating	______________________________
